# Photobleaching studies reveal that a single amino acid polymorphism is responsible for the differential binding affinities of linker histone subtypes H1.1 and H1.5

**DOI:** 10.1242/bio.016733

**Published:** 2016-02-24

**Authors:** Thomas W. Flanagan, Jacob K. Files, Kelsey Rose Casano, Eric M. George, David T. Brown

**Affiliations:** 1 Department of Biochemistry, University of Mississippi Medical Center, Jackson, MS 39216, USA; 2 Clinton High School, Clinton, MS 39056, USA; 3 Spring Hill College, Mobile, AL 36608, USA; 4 Saint Andrew's Episcopal School, Ridgeland, MS 39157, USA; 5 Department of Physiology, University of Mississippi Medical Center, Jackson, MS 39216, USA

**Keywords:** Histone H1, Linker histone, Chromatin, FRAP

## Abstract

Mammals express six major somatic linker histone subtypes, all of which display dynamic binding to chromatin, characterized by transient binding at a given location followed by rapid translocation to a new site. Using photobleaching techniques, we systematically measured the exchange rate of all six mouse H1 subtypes to determine their relative chromatin-binding affinity. Two subtypes, H1.1 and H1.2, display binding affinities that are significantly lower than all other subtypes. Using *in vitro* mutagenesis, the differences in chromatin-binding affinities between H1.1 (lower binding affinity) and H1.5 (higher binding affinity) were mapped to a single amino acid polymorphism near the junction of the globular and C-terminal domains. Overexpression of H1.5 in density arrested fibroblasts did not affect cell cycle progression after release. By contrast, overexpression of H1.1 resulted in a more rapid progression through G1/S relative to control cells. These results provide structural insights into the proposed functional significance of linker histone heterogeneity.

## Abstract (precis)

**Summary:** Mouse linker histone subtypes H1.1 and H1.5 bind to chromatin with different affinities due to a single amino acid polymorphism. Overexpression of H1.1 in fibroblasts accelerates cell cycle progression.

## INTRODUCTION

The fundamental repeating unit of eukaryotic chromatin is the nucleosome (Kornberg and Thomas, 1974; Wolffe, 1998). The nucleosome core particle is composed of 147 bp of DNA wrapped around an octamer of two molecules each of the core histones H2A, H2B, H3 and H4 (Luger et al., 1997; Woodcock, 2006). In metazoans, approximately one molecule of the linker or H1 is bound to DNA at the entry/exit site on the surface of the nucleosomal core to form the chromatosome (Simpson, 1978; Thoma et al., 1979) and also associates with additional linker DNA between adjacent nucleosomes to promote chromatin condensation into higher-order structures (Bednar et al., 2016, 1998; Robinson and Rhodes, 2006; Woodcock et al., 2006). In higher organisms, the linker histones have a conserved tripartite structure composed of a short, flexible N-terminal tail (∼45 residues) enriched in basic amino acids, a highly conserved central globular domain (∼80 residues) consisting of a three helix bundle containing a winged-helix fold, and a long, extremely lysine-rich C-terminal tail (Allan et al., 1980; Ramakrishnan et al., 1993; Roque et al., 2016). The C-terminal tail is necessary for H1-linker DNA binding and chromatin stabilization and condensation (Allan et al., 1986; Georgel and Hansen, 2001; Thoma et al., 1979), whereas the globular domain confers nuclease protection to the chromatosome (Noll and Kornberg, 1977) and influences the structural geometry of condensed chromatin (Bednar et al., 2016; Cutter and Hayes, 2015; Roque et al., 2016; Vyas and Brown, 2012).

In mammalian species the H1 histones exist as a family of ten or more non-allelic primary sequence subtypes, or subtypes, and there is considerable evidence that there is a functional significance to this heterogeneity (Happel and Doenecke, 2009; Hergeth and Schneider, 2015; Izzo et al., 2008; Khochbin and Wolffe, 1994; Lennox and Cohen, 1983; Millan-Arino et al., 2016; Parseghian and Hamkalo, 2001). Most mouse somatic tissues express six major subtypes. The replication-dependent subtypes, H1.1 through H1.5, are expressed primarily during the S phase of the cell cycle. These subtypes have a highly conserved central globular domain but display considerable sequence variation in the terminal domains. A sixth somatic subtype, H1.0, is expressed throughout the cell cycle, accumulates in terminally differentiated cells, and displays considerable sequence divergence from the other H1 subtypes in the globular as well as the terminal domains (Zlatanova and Doenecke, 1994). Knockout mice lacking any one of the main H1 subtypes display no discernible phenotypic change, as the remaining genes compensate to produce a normal H1-to-nucleosome stoichiometry (Fan et al., 2001). However, when multiple linker histone genes are inactivated, there is a reduced H1-to-nucleosome ratio and overall changes to chromatin organization, resulting in embryonic lethality (Fan et al., 2003). In cultured cells, knockdown or overexpression of individual linker histone subtypes has differing effects on chromatin structure, gene expression and cell cycle progression (Bhan et al., 2008; Brown et al., 1996; Gunjan et al., 1999; Sancho et al., 2008).

Results from photobleaching experiments demonstrate that linker histones interact dynamically with chromatin *in vivo* (Flanagan and Brown, 2016; Lever et al., 2000; Misteli et al., 2000). At any moment, the vast majority of H1 molecules are bound to chromatin, but this binding is transient, lasting approximately one minute before the H1 dissociates and moves to another site. This continuous exchange has led to the postulation that H1 might function along with a network of interacting factors to modulate chromatin structure and function via transient localized decondensation (Catez et al., 2004; Postnikov and Bustin, 2016).

Because there is a negligible pool of unbound linker histones and the exchange rate is not diffusion limited, kinetic parameters determined by fluorescence recovery after photobleaching (FRAP) represent a quantitative measure of the *in vivo* binding affinities of these proteins to nucleosomes (Flanagan and Brown, 2016). Our lab has used a systematic approach involving point mutations and FRAP to map the position of the globular domain of H1.0 onto the chromatosome (Brown et al., 2006), to demonstrate differences in binding orientation between H1.0 and H1.2 (George et al., 2010), and to identify contributions of the N-terminal domains of linker histones chromatin-binding affinity (Vyas and Brown, 2012). In this study we have utilized a systematic approach to obtain kinetic information for all six of the major somatic H1 subtypes of mouse. We then focused on two subtypes, H1.1 and H1.5 as recent studies suggest that these ‘minor’ subtypes are uniquely distributed throughout the genome and may have specific functions in organizing chromatin structure (Izzo et al., 2013; Li et al., 2012; Millan-Arino et al., 2016; Terme et al., 2011). Using *in vitro* mutagenesis, we identified a single amino acid polymorphism near the junction of the globular and C-terminal domains that is responsible for the differences in chromatin-binding affinities between these subtypes. We also report that overexpression of H1.1 results in accelerated progression through G1/S upon release of synchronized cells from density arrest.

## RESULTS

### Individual H1 subtypes display distinct chromatin-binding properties

**Fig. 1.**
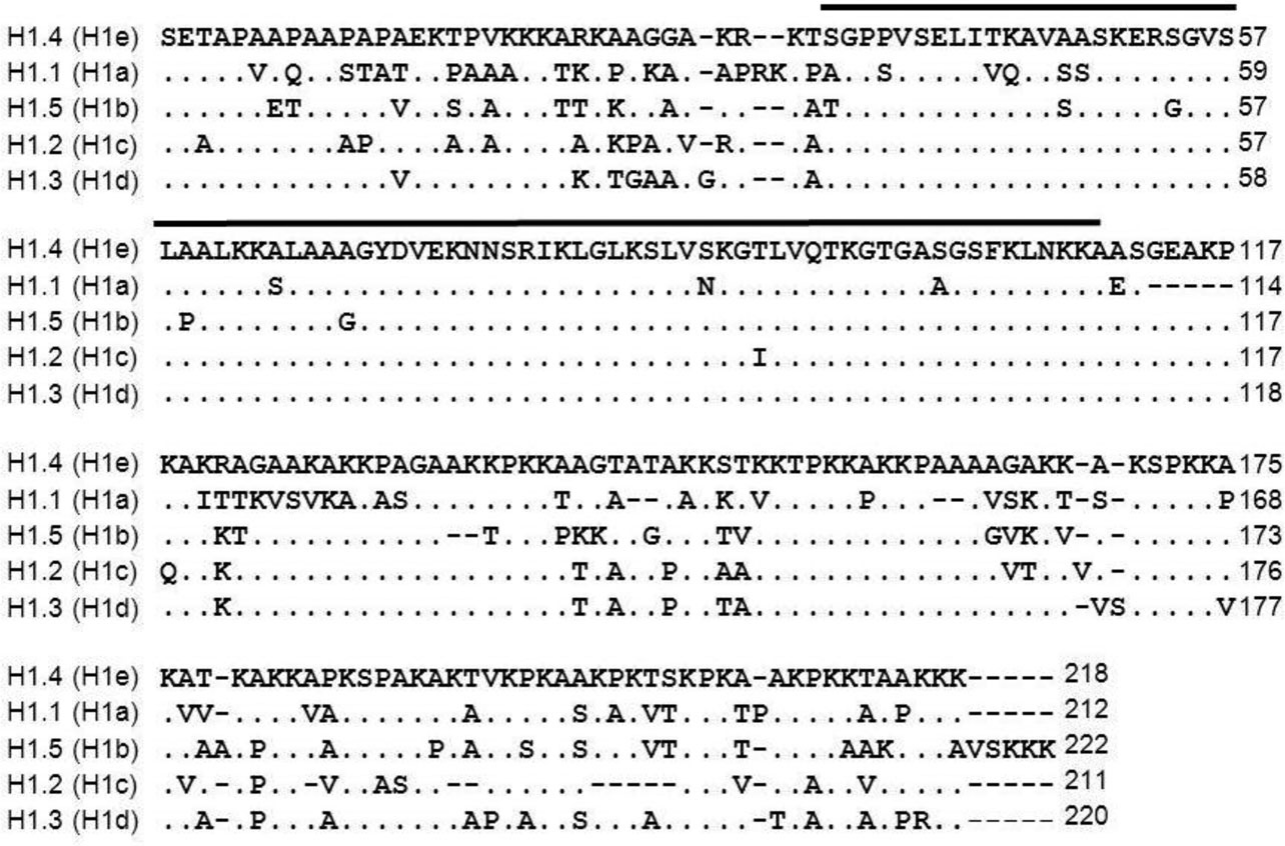
**Sequence comparison of *Mus musculus* replication-dependent somatic linker histone subtypes.** The following sequences were aligned to H1.4 (Genbank accession no. NM_015787) using BLOSUM62: H1.1 (Genbank accession no. NM_030609), H1.5 (Genbank accession no. NM_020034), H1.2 (Genbank accession no. NM_015786), H1.3 (Genbank accession no. NM_145713). Subtypes are designated according to the recently proposed unified nomenclature (Talbert et al., 2012). The previously used mouse subtype designations are shown in parentheses. The sequence span representing the globular domain is marked with a black bar.

The six major mouse somatic H1 subtypes maintain a similar tripartite domain structure but display evolutionarily conserved sequence variations (Happel and Doenecke, 2009; Izzo et al., 2008; Parseghian and Hamkalo, 2001; Talbert et al., 2012) (Fig. 1). For the replication-dependent subtypes, H1.1-H1.5, the sequence divergence is mainly in the N- and C-terminal domains. The replication-independent H1.0 subtype differs from the other subtypes within the globular domain as well.

**Fig. 2.**
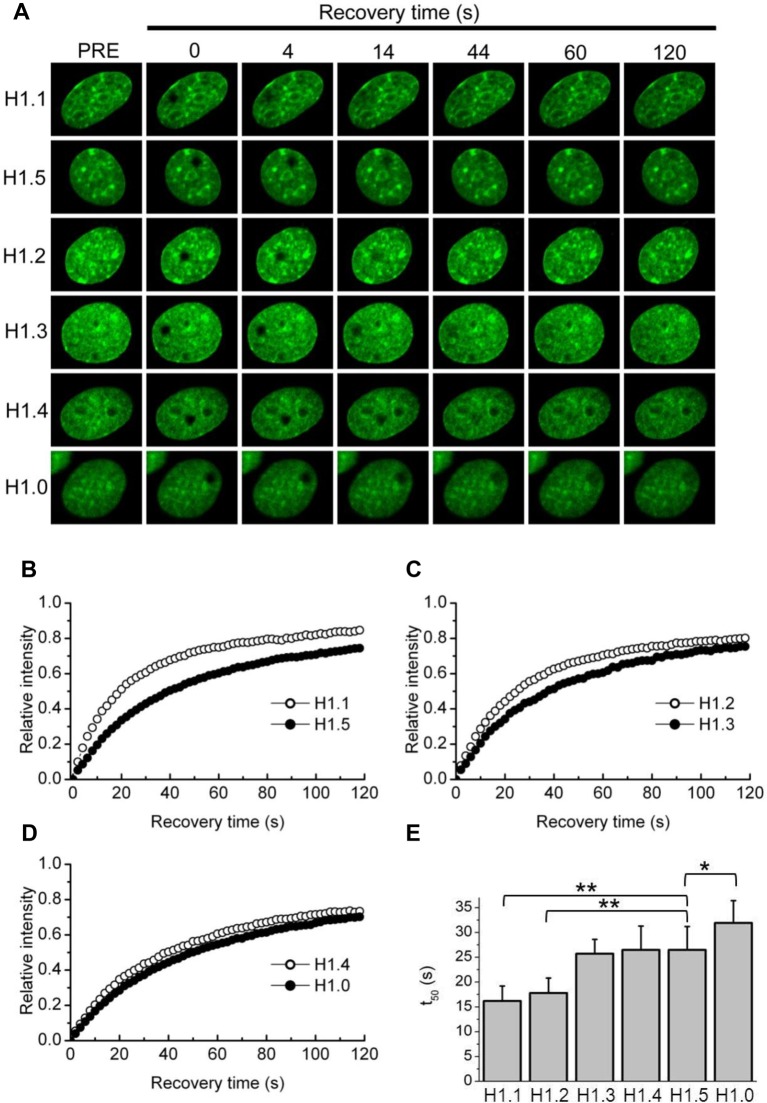
**FRAP analysis of individual H1 subtypes.** (A) Representative FRAP analyses demonstrating the kinetic properties of each of the somatic linker histone subtypes. BALB/c 3T3 cells stably expressing H1-GFP proteins were imaged before and during recovery after bleaching of a nucleoplasmic area 2 µm in diameter. Images were taken before (pre-bleach, left column) and at indicated times after the bleach pulse. (B-D) Quantitative analysis of FRAP recovery of cells expressing the indicated H1-GFP subtypes. Error bars have been removed for clarity. (F) Histogram of t_50_ values. Plotted values represent means±s.d. from at least 12 measurements (see Table 1); **P<0.0001; *P<0.001.

We created a series of expression vectors in which the coding sequence of each of the somatic subtypes was fused to the amino terminus of EGFP. Constructs were stably transfected into mouse fibroblasts and individual cell lines expressing low levels of the exogenous construct were isolated and visualized by confocal microscopy (Fig. 2A). Non-random subnuclear distribution of individual H1 subtypes has been reported in human and mouse cells (Millan-Arino et al., 2016; Parseghian et al., 2000; Th'ng et al., 2005). By observation, we noted no obvious or consistent differences among the subtypes in the morphology of the cells or nuclei or in the sub-nuclear distribution of the subtypes. All six subtypes were underrepresented in nucleoli, enriched in heterochromatin and co-localized with DNA as determined by Hoechst 33342 binding (Misteli et al., 2000) (data not shown).

We performed FRAP analysis on exponentially growing cultures of each of these cell lines to quantitatively measure binding parameters (Fig. 2, Table 1). H1 binding, as measured by FRAP analysis, is composed of at least two kinetic classes (Misteli et al., 2000; Raghuram et al., 2010). In this study we limited the recovery time to two minutes to specifically measure the rapidly exchanging fraction. This class, sometimes referred to as the low affinity fraction, comprises ∼75% or more of the total population of any given subtype. The recovery curves all fit well to single exponential binding (R^2^>0.99) and we report binding parameters as the half-time of recovery or t_50_ values (Fig. 2E, Table 1). We also report the immobile fraction, which is an estimation of the amount of the total population engaged in high affinity binding interactions and therefore does not recover within two minutes.

**Table 1.**
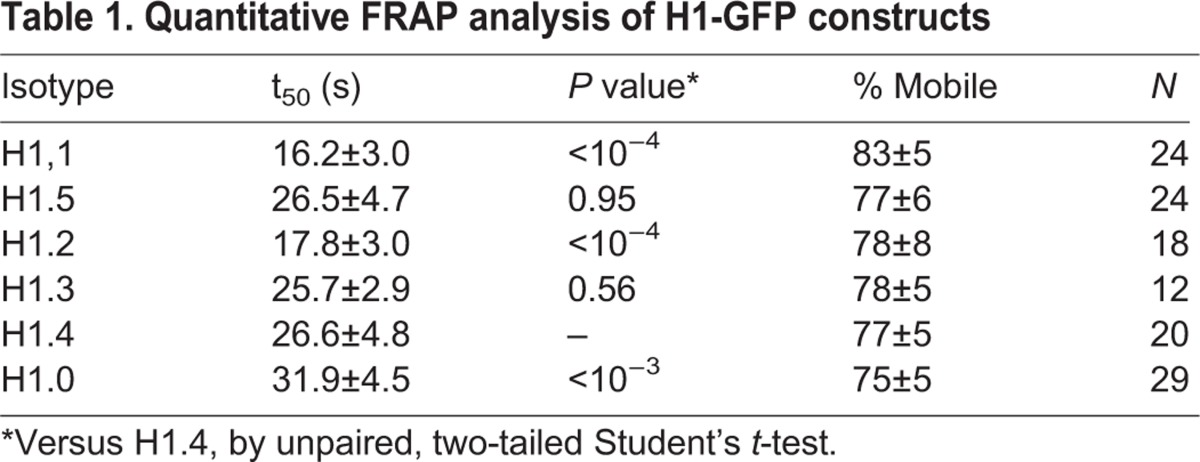
**Quantitative FRAP analysis of H1-GFP constructs**

Subtypes H1.3, H1.4, and H1.5 exhibited similar recovery kinetics with t_50_ values of ∼26 s (Fig. 2, Table 1). The replication-independent H1.0 subtype exhibited the slowest recovery, i.e. the strongest affinity as has previously been reported (George et al., 2010). The H1.2 subtype displayed a significantly faster recovery (t_50_=18 s) which is also consistent with previous reports. Interestingly, the H1.1 subtype displayed the fastest recovery (t_50_=∼16 s) and a much smaller immobile fraction than all other subtypes, suggesting that the binding properties of this minor H1 subtype are distinct from most other somatic subtypes.

### Identification of a single amino acid polymorphism responsible for the differential binding affinities of H1.1 and H1.5

**Fig. 3.**
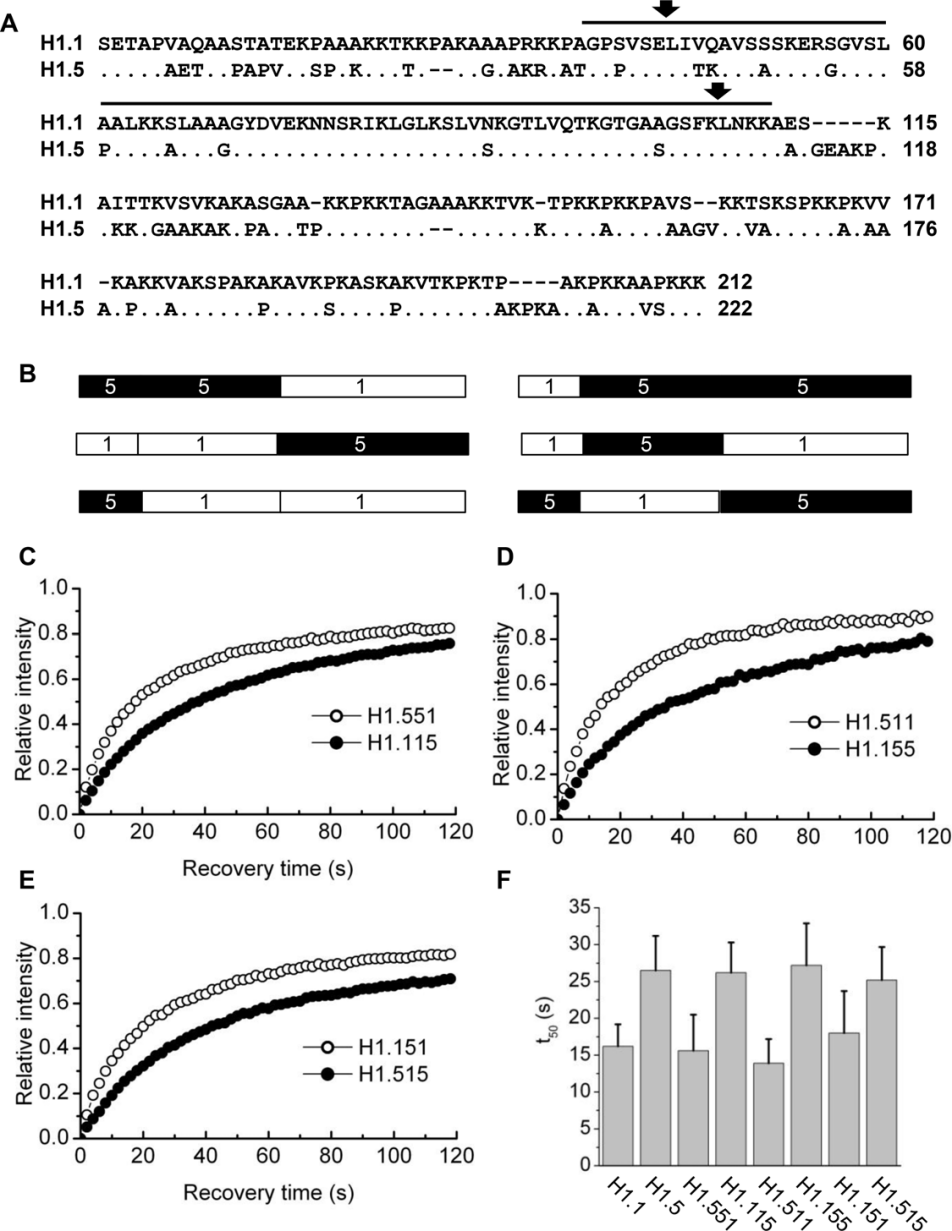
**Quantitative analysis of FRAP recovery of domain switch constructs.** (A) Sequence alignment of H1.1 and H1.5. The sequence span representing the globular domain is marked with a black bar. Arrows represent the location of the complimentary *Sac*1 and *Hind*3 restriction sites used to swap domains between the two variants. (B) Schematic of domain switch mutants. Construct 551 consists of residues 1-99 of H1.5 and residues 102-213 of H1.1; Construct 115 consists of residues 1-101 of H1.1 and residues 100-223 of H1.5. Construct 511 consists of residues 1-42 of H1.5 and residues 45-213 of H1.1. Construct 155 consists of residues 1-44 of H1.1 and residues 43-223 of H1.5. Construct 151 consists of residues 1-44 of H1.1, residues 43-99 of H1.5 and residues 102-213 of H11. Construct 515 consists of residues 1-42 of H1.5, residues 45-101 of H1.1 and residues 100-223 of H1.5. (C-E) Quantitative analysis of FRAP recovery of cells expressing the indicated domain switch constructs as GFP fusions. (F) Plotted t_50_ values represent means±s.d. from at least 12 cells (see Table 2).

To gain insight into the structural basis of the low chromatin-binding affinity of the H1.1 subtype, we created a series of domain switch constructs using the H1.1 and H1.5 subtypes. Within the coding region of the genes for these proteins are identical restriction enzyme sites located in codons for conserved amino acids. One of these is located near the junction of the N-terminus and the globular domain, and we engineered another near the junction of the globular domain and the C-terminus (Fig. 3A), allowing us to make all possible domain switch combinations (Fig. 3B). These constructs were expressed in mouse fibroblasts and the chromatin-binding parameters of each protein were determined using FRAP analysis (Fig. 3C-E, Table 2). Analysis of the results revealed that the difference in the chromatin-binding affinity of these two subtypes clearly segregates with the C-terminal domain. All constructs containing the H1.1 C-terminal domain, regardless of the origin of the other two domains, recovered faster than the reciprocal constructs containing the H1.5 C-terminal domain (Fig. 3F). Interestingly, the lower amounts of the statically bound or immobile fraction observed for H1.1 also segregated with the C-terminal domain.

**Table 2.**
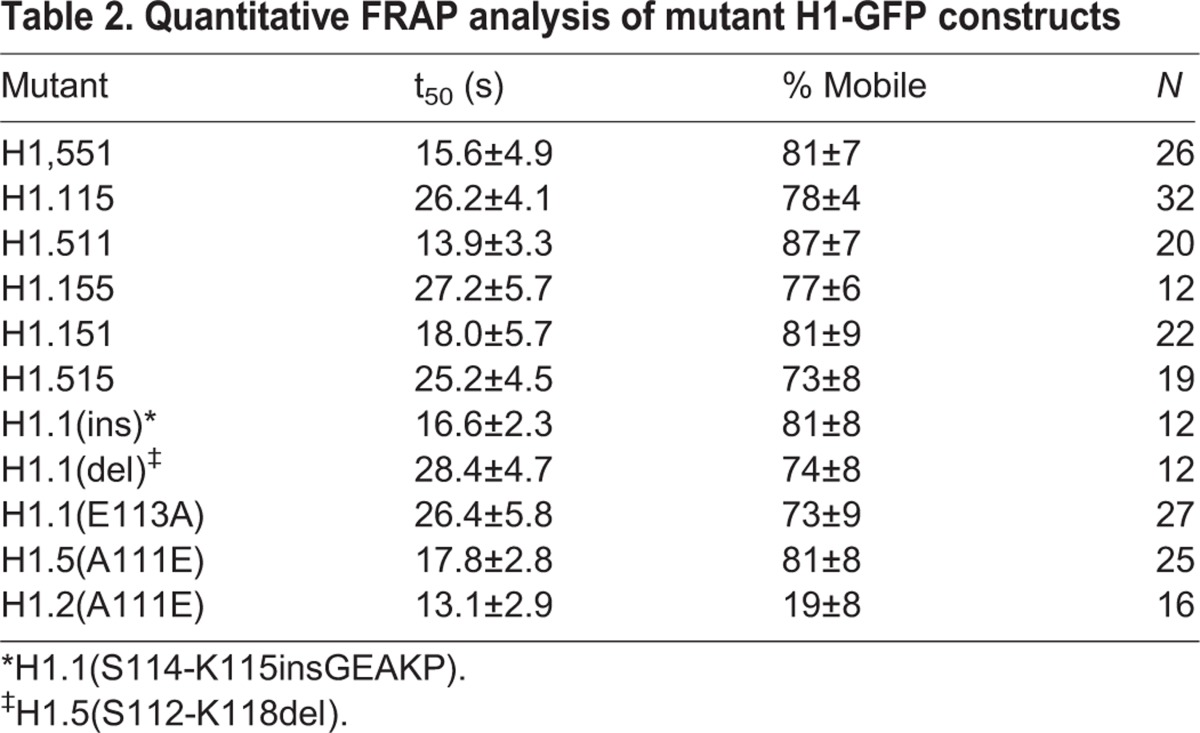
**Quantitative FRAP analysis of mutant H1-GFP constructs**

**Fig. 4.**
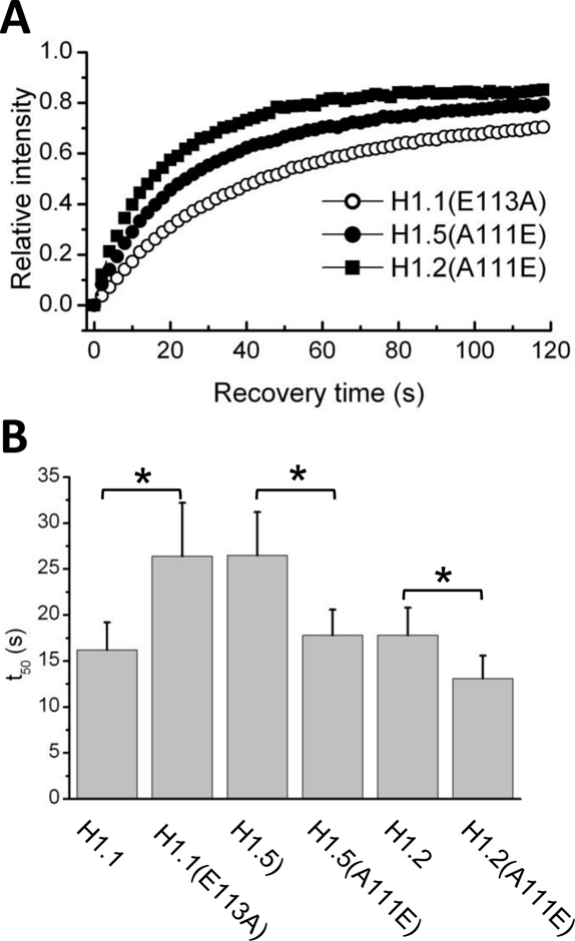
**Quantitative analysis of FRAP recovery of mutant H1 constructs.** (A) Quantitative analysis of FRAP recovery of cells expressing the indicated constructs as GFP fusions. (B) Plotted t_50_ values represent means±s.d. from at least 16 cells (see Table 2). *P<0.0001.

By inspection of the sequences of the replication-dependent subtypes (Figs 1 and 3A) we noted that a highly conserved sequence (GEAKP) located in the C-terminus of subtypes H1.2-H1.5 near the junction with the globular domain, is conspicuously absent from H1.1. We considered that the absence of this sequence might contribute to the lower binding affinity of H1.1. We created a construct, H1.1(ins) in which we inserted sequences encoding GEAKP between codons 114 and 115 of H1.1. We also created the reciprocal construct, H1.5(del) in which the sequences were deleted. However, FRAP analysis of these constructs revealed that insertion of these sequences into H1.1 or deletion from H1.5 did not affect the *in vivo* binding parameters (Table 2). From further inspection of the sequences we noted that there is an alanine residue at position 111 of H1.5 that is conserved in all other replication-dependent variants except H1.1, in which the residue in the same position is glutamic acid. We therefore created and analyzed reciprocal constructs with point mutations in these residues, i.e. H1.1(E113A) and H1.5(A111E). Remarkably, mutations at this single position have dramatic effects on the *in vivo* binding properties of these proteins (Fig. 4). The t_50_ of the H1.1(E113A) construct was nearly identical to that of H1.5 (∼26 s). By contrast, the A111E mutation in H1.5 resulted in a t_50_ of 17.8 s, almost the same value as H1.1. We conclude that the amino acid polymorphism at this site is a major determinant of the differential binding affinities of these two variants.

Curiously, the H1.2 variant displays a binding affinity that is similar to that of H1.1 but possesses an alanine residue at position 111. We therefore created and analyzed an A111E mutant of H1.2 (Fig. 4). This mutation further reduced the t_50_ value to ∼13 s. We had previously demonstrated through domain swapping mutagenesis that the differences between the binding affinities of H1.2 and H1.0 were attributable to sequences in the N-terminal domains (Vyas and Brown, 2012). We conclude that the lower binding affinities of H1.1 and H1.2 relative to the other variants are mediated by distinct structural components.

The location of the GFP tag has been reported to influence the quantitative FRAP recovery times of H1 subtypes (Hendzel et al., 2004). We therefore constructed and analyzed cell lines expressing H1.1 and H1.5 tagged with GFP at the N-terminus (Fig. S1A). These constructs did recover slightly slower than the corresponding construct with a C-terminal tag but GFP-H1.1 still recovered significantly faster than GFP-H1.5. Furthermore, the GFP-H1.1(E113A) and GFP-H1.5(A111E) mutations essentially reversed the relative recovery times as was observed for the C-terminally tagged mutants (Fig. S1B).

### Overexpression of H1.1 accelerates G1 and S-phase progression

**Fig. 5.**
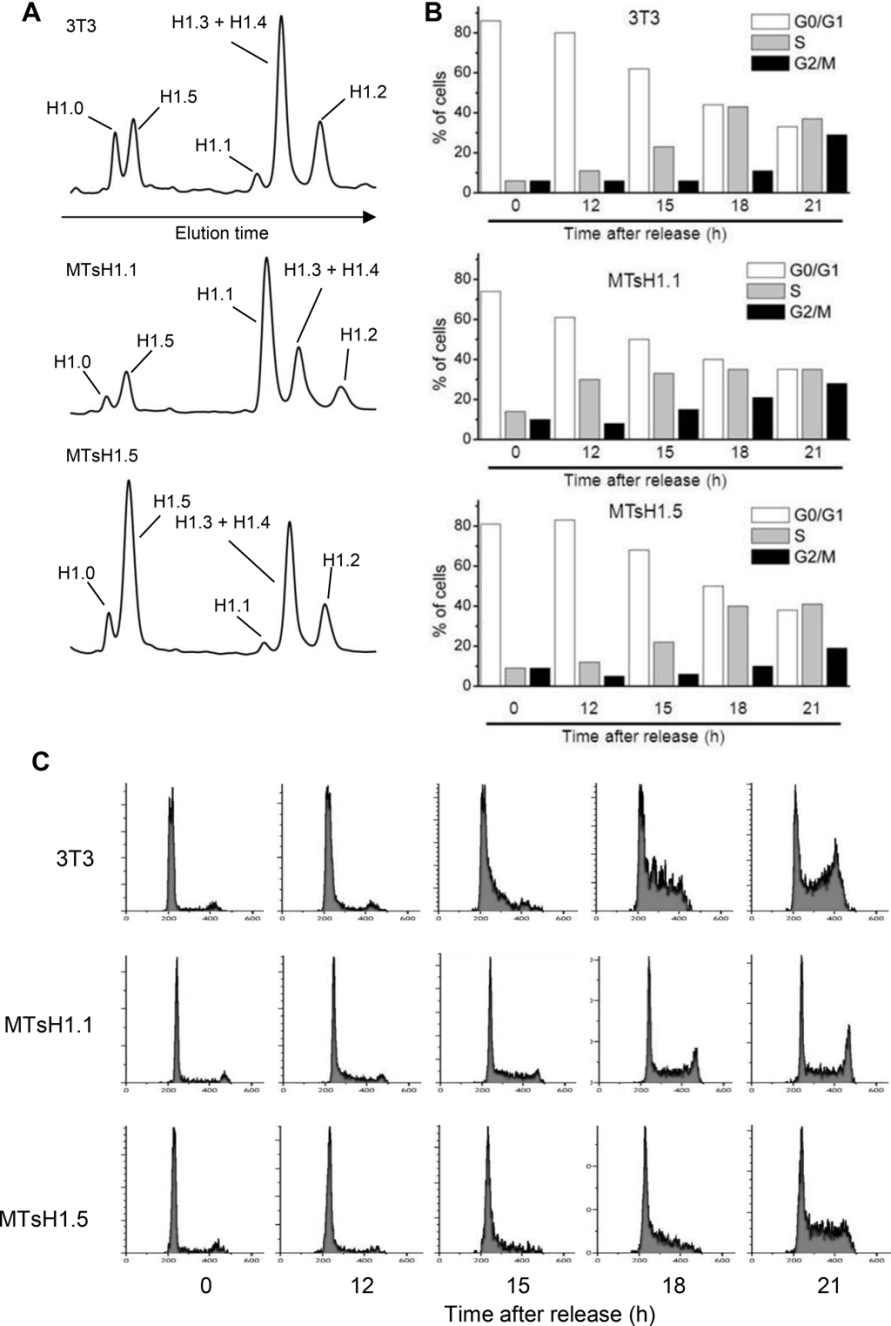
**Effects of overexpression of H1.1 or H1.5 on cell cycle progression.** (A) Separation of H1 subtypes by HPLC. Total histones were isolated from density arrested cell cultures from the indicated lines and separated by HPLC as previously described (Brown et al., 1996). The subtypes present in each peak are indicated based on prior studies (Yellajoshyula and Brown, 2006). Estimates of the relative amounts of each subtype were determined by quantitation of the absorbance at 210 nm via integration of the area in each peak (see Table 3). (B) Cell cycle distribution after release from density arrest as determined by FACS. Results are the average of three independent experiments. (C) Individual FACS profiles from a representative experiment.

We have previously demonstrated that forced overexpression of the H1.0 subtype significantly slows progression through G1 and S-phases of the cell cycle while overexpression of the H1.2 variant to similar levels was without effect (Brown et al., 1996). We have since attributed the differences between these responses to overexpression, in part to the observed differences between these variants in their chromatin binding affinities (George et al., 2010). We were interested then in determining the effect of overexpression of H1.1 and H1.5 on cell cycle progression. The coding regions for these proteins, lacking GFP tags, were cloned into an expression vector such that they are under the transcriptional control of the heavy metal-inducible mouse metallothionein promoter. We also replaced the stem-loop sequences found in the 3′ UTR of these replication-dependent genes with sequences containing a polyadenylation-directing site so that the resulting mRNA would be stable outside of S-phase. We transfected these plasmids into 3T3 fibroblasts and isolated stable cell lines that overexpressed either H1.1 or H1.5. These cell lines, as well as control 3T3 cells, were plated on 150 cm^2^ dishes an allowed to reach confluency. Increasing concentrations of ZnCl_2_ were added to the culture medium for the next 48 h to induce expression from the exogenously introduced constructs. Forty eight hours after reaching confluency, total histones were isolated from one flask and separated by HPLC (Fig. 5A, Table 3). The results indicate that the appropriate cell lines have accumulated H1.1 or H1.5 to comparable levels and that levels of the other variants are reduced by an apparent compensatory response as has been previously observed (Brown et al., 1996). Cells from a second flask were harvested by trypsinization, diluted 10-fold with fresh medium and re-plated. Aliquots were removed at intervals following release and cell progression was monitored by FACS (Fig. 5B,C). The cell line overexpressing H1.5 re-entered the cell cycle with similar kinetics to control; cells began to appear in S-phase at 15 h and in G2/M at 21 h after release from density arrest (Fig. 5B). By contrast, the H1.1-overexpressing cell line appeared to enter S-phase earlier with a significant increase at 12 h. These cells also appeared to progress through S-phase slightly faster than control or H1.5 overexpressing cells (Fig. 5C). At 15 and 18 h after release, cultures over-expressing H1.1 are predominantly in mid-S to late-S-phase while control and H1.5 over-expressing cultures are predominantly in early-S or mid-S-phase.

**Table 3.**
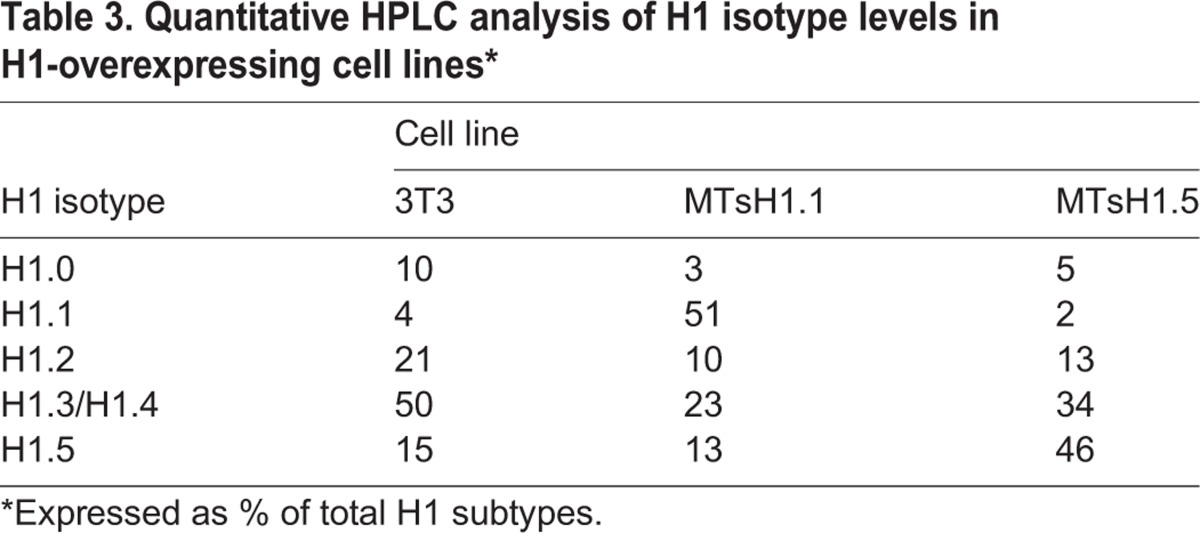
Quantitative HPLC analysis of H1 isotype levels in H1-overexpressing cell lines*

## DISCUSSION

This study was initiated to generate a compilation of quantitative chromatin-binding parameters for all of the major somatic mouse H1 histone subtypes using identical vectors, cultured cells, and confocal instrumentation. Similar comprehensive analyses have been performed with the human orthologs (Raghuram et al., 2010; Th'ng et al., 2005), but previous work in the mouse has focused primarily on H1.0 and H1.2 (Brown et al., 2006; George et al., 2010; Vyas and Brown, 2012). Comprehensive information about the murine subtypes is valuable as many functional studies of H1 heterogeneity utilize mouse-based systems, most notably knockout and more recently knock-in approaches (Cao et al., 2013; Fan et al., 2003, 2005; Yang et al., 2013). The results from these recent reports have significantly strengthened the case for a functional significance to linker histone heterogeneity in the mouse. While the results obtained in this study broadly agree with those using human cells (Raghuram et al., 2010; Th'ng et al., 2005), our experimental design differs from those studies notably in that we are focusing specifically on the rapidly exchanging population. Furthermore, there are potentially significant differences between human and mouse orthologs; for example, the mouse human H1.1 orthologs are only 78% identical (Talbert et al., 2012).

From our photobleaching studies, we conclude that, at least in fibroblasts, three of the six subtypes tested, H1.1, H1.2 and H1.0, displayed kinetic behavior distinct from that of the other subtypes. Notably, the recovery of the H1.1 and H1.2 subtypes was significantly faster, indicating that they are exchanging more rapidly, an observation that is generally interpreted to reflect reduced binding affinity (Flanagan and Brown, 2016). In addition, we find that a much smaller fraction of the H1.1 subtype population is engaged in static binding. It should be noted that we purposefully avoided photobleaching H1-dense chromocenters as we believe these may consist of a high percentage of constitutive heterochromatin. We have instead focused on regions of the nucleus that most likely contain both euchromatin and heterochromatin and attempted to make a significant number of measurements to avoid bias. We feel that it is an open question as to whether static binding can be strictly interpreted as a property of heterochromatin.

In our previous studies we were able to utilize results from mutagenesis and photobleaching assays to draw conclusions regarding the binding orientation of the globular domain of the H1.0 and H1.2 subtypes to chromatosomal DNA (Brown et al., 2006; George et al., 2010). We concluded that the binding interfaces of these two subtypes are different, implying that they bind to nucleosomes with distinct orientations. A subsequent domain swap study lead to the surprising conclusion that differences between these subtypes in the N-terminal domain were responsible for the differences in the overall binding affinity (Vyas and Brown, 2012).

Here we conducted a similar strategy using the low affinity binding H1.1 subtype and the stronger binding H1.2 subtype. The results clearly identified the C-terminus as the domain responsible for the difference in the binding affinity between these two subtypes. Through additional mutagenesis, we made the surprising observation that polymorphism of a single amino acid residue, Glu-111 of H1.1 and Ala-113 of H1.5, located at the junction of the globular and C-terminal domains, is responsible for the very different binding affinities of these two variants. While it is not immediately obvious why this residue is crucial for binding of linker histones to the nucleosome, several recent biophysical studies indicate that sequences near the junction of the globular and C-terminal domains are critical for proper positioning of the linker histone within the nucleosome (Fang et al., 2012; Lu and Hansen, 2004; Syed et al., 2010; Zhou et al., 2013). The observation presented here will be useful as structural information on the nucleosomal binding of H1 continues to accrue (Zhou et al., 2015).

We observed that overexpression of H1.5 in density arrested fibroblasts did not significantly impact cell cycle progression upon subsequent release. Interestingly, overexpression of H1.5 results in a significant decrease in the amount of H1.2 bound to chromatin (Fig. 5A). Knockdown of H1.2 in a human breast cancer cell resulted in a strong cell cycle arrest in G1 (Sancho et al., 2008). It is possible that H1.5 has specific overlapping functions with H1.2 that compensate for the reduced amounts of the latter. We also observed that overexpression of H1.1 resulted in an accelerated progression through G1 and S-phase. Several reports indicate that chromatin de-condensation involving H1 removal is a necessary event for DNA replication (Alexandrow and Hamlin, 2005; De et al., 2002; Lu et al., 1997). While it is possible that the lower binding affinity of H1.1 facilitates this process it is also conceivable that, as described below, unique interactions of H1.1 with chromatin may contribute as well.

We focused on the H1.1 and H1.5 subtypes in part because they are quantitatively minor variants in most tissues and have thus received less attention, but also because several interesting reports regarding these variants have recently appeared. The DNA-binding protein BAF was reported to bind specifically to the C-terminal domain of the human H1.1 subtype (Montes de Oca et al., 2005). Interestingly, a recent study of the genomic distribution of human H1 subtypes found that H1.1 differed significantly from the other somatic subtypes in many aspects (Izzo et al., 2013). Notably, unlike the other subtypes, H1.1 was not depleted from active and poised promoters. Human H1.5 was shown to be differentially distributed throughout the genome in differentiated cells versus embryonic stem cells (Li et al., 2012). Analysis of data from a number of approaches leads to the view that chromatin is heterogeneous and that quantitative and qualitative differences in the binding of linker histones to the nucleosome may contribute to the presence of different forms of higher order chromatin structure (Ausió, 2015; George et al., 2010; Grigoryev et al., 2009; Zhou et al., 2015). The results presented here are consistent with that view.

## MATERIALS AND METHODS

### Plasmid constructs and cell lines

Plasmids for the expression of H1.0 and H1.2 with C-terminal EGFP tags were previously described (Misteli et al., 2000). As the genes for H1.1, H1.3, H1.4 and H1.5 lack introns, the coding regions for these genes were PCR amplified directly from mouse genomic DNA using subtype-specific primers carrying additional sequences to allow the amplicon to be directly cloned into expression vectors. In these plasmids, the coding sequence for enhanced GFP is fused to the C terminus of the coding region for the histone, and expression is under control of the mouse metallothionein promoter. Constructs were transfected into mouse BALB/c 3T3 cells propagated in DMEM-low glucose supplemented with 10% heat inactivated bovine serum. Multiple stable transfectants were isolated and analyzed as described below. For the domain swap constructs we took advantage of *Sac*1 restriction sites that span conserved codons at positions 44-45 of H1.1 and 42-43 of H1.5 and unique *Hind*3 restriction sites that were engineered into conserved codons at positions 107-108 of H1.1 and 105-106 of H1.5. Specific deletion/insertion mutations and point mutations were introduced using the Q5^®^ Site-Directed Mutagenesis Kit (New England Biolabs, Inc.) following the manufacturer's instructions. In these plasmids expressing mutant H1-GFP, expression is under control of the CMV promoter. Plasmids for the expression of H1.1 and H1.5 with an N-terminal GFP tag were constructed as previously described (Vyas and Brown, 2012). Plasmids for the overexpression of untagged H1.1 and H1.5 proteins (MTsH1.1, MTsH1.5) were constructed by PCR-amplification of the coding region and insertion into expression vectors under control of the mouse metallothionein promoter as previously described (Brown et al., 1996). Expression vectors were transfected into mouse BALB/c 3T3 cells propagated in DMEM-low glucose supplemented with 10% heat inactivated bovine serum. Stable transformants were isolated in the presence or 3 µg/ml of puromycin and overexpressing cell lines were identified by isolation and fractionation of total histones by HPLC as previously described (Brown et al., 1996).

### FRAP assays

For FRAP assays, cultures were grown in 35 mm glass bottom microwell dishes (MatTek Corporation). For initial studies comparing the kinetics of individual H1 subtypes (Fig. 2) stable cell lines were grown in the absence of the inducer ZnCl_2_ to yield low level constitutive expression (Misteli et al., 2000). Under these conditions, H1-GFP comprises less than 5% of the total H1 population. For studies of mutant H1 constructs, plasmids were transiently transfected into 3T3 cells and FRAP assays were performed 48 h later. Only cells expressing low levels of H1-GFP were analyzed. Control experiments demonstrated that transient and stable transfection procedures gave identical results (data not shown). FRAP was performed on a Leica TCS SP8 laser scanning confocal microscope using the 488 nm line of an argon laser. All experiments were carried out at 37°C, and imaging was performed with a Plan Apo 63/1.3 objective lens using the FRAP module of the Leica LAS AF software. For each experiment, three pre-bleach images were taken, and a single 2 μm spot was bleached with the 488 nm line at 100% transmission. Post-bleach scanning was bidirectional at 400 mHz for 120 s at 2 s intervals using a ×4 zoom with a pinhole of 2 Airy units. Image analysis was carried out within the LAS AF software. Quantitative data were imported into easyFRAP (Rapsomaniki et al., 2012), double normalized and fitted to a single exponential curve. All datasets consisted of at least 12 cells per experiment.

### Cell cycle analysis

Control cells and cell lines overexpressing H1.1 or H1.5 were allowed to grow to confluency then treated for 48 h with ZnCl_2_. Cultures were then trypsinized, diluted 10-fold and re-plated into fresh medium. Aliquots were harvested prior to and at intervals following re-plating. Cells were washed with cold PBS and fixed in 5 ml of cold 90% methanol with gentle agitation and stored at −20°C until further use. After fixation, cells were washed, pelleted, and resuspended in PBS. RNase A (Sigma) was added to a final concentration of 850 µg/ml for 5 min, followed by 85 µg/ml propidium iodide (PI, Sigma) for 30 min at room temperature. Samples were analyzed on a Gallios flow cytometer at the University of Mississippi Medical center Flow Cytometry Core Facility. 10,000 events were collected and the data was analyzed using Kaluza software (Beckman Coulter).
